# The burden of neck pain: its meaning for persons with neck pain and healthcare providers, explored by concept mapping

**DOI:** 10.1007/s11136-015-1149-6

**Published:** 2015-10-14

**Authors:** Carlijn H. van Randeraad-van der Zee, Anna J. H. M. Beurskens, Raymond A. H. M. Swinkels, Jan J. M. Pool, Roy W. Batterham, Richard H. Osborne, Henrica C. W. de Vet

**Affiliations:** Department of Epidemiology and Biostatistics, EMGO+ Institute for Health and Care Research, VU University Medical Center, Boelelaan 1089A, 1081 HV Amsterdam, The Netherlands; Centre of Research Autonomy and Participation for Persons with a Chronic Illness, Zuyd University of Applied Sciences, Heerlen, The Netherlands; Department of Family Practice, Caphri, Maastricht University, Maastricht, The Netherlands; Department of Physical Therapy, Zuyd University of Applied Sciences, Heerlen, The Netherlands; Research Center for Innovations in Healthcare, University of Applied Science Utrecht, Utrecht, The Netherlands; Public Health Innovation, Population Health Strategic Research Centre, School of Health and Social Development, Deakin University, Burwood, Australia

**Keywords:** Concept mapping, Burden of neck pain, Patient-reported outcomes, Multidimensional scaling, Cluster analysis, Content validity, Mind map

## Abstract

**Purpose:**

To empirically define the concept of burden of neck pain. The lack of a clear understanding of this construct from the perspective of persons with neck pain and care providers hampers adequate measurement of this burden. An additional aim was to compare the conceptual model obtained with the frequently used Neck Disability Index (NDI).

**Methods:**

Concept mapping, combining qualitative (nominal group technique and group consensus) and quantitative research methods (cluster analysis and multidimensional scaling), was applied to groups of persons with neck pain (*n* = 3) and professionals treating persons with neck pain (*n* = 2). Group members generated statements, which were organized into concept maps. Group members achieved consensus about the number and description of domains and the researchers then generated an overall mind map covering the full breadth of the burden of neck pain.

**Results:**

Concept mapping revealed 12 domains of burden of neck pain: impaired mobility neck, neck pain, fatigue/concentration, physical complaints, psychological aspects/consequences, activities of daily living, social participation, financial consequences, difficult to treat/difficult to diagnose, difference of opinion with care providers, incomprehension by social environment, and how person with neck pain deal with complaints. All ten items of the NDI could be linked to the mind map, but the NDI measures only part of the burden of neck pain.

**Conclusion:**

This study revealed the relevant domains for the burden of neck pain from the viewpoints of persons with neck pain and their care providers. These results can guide the identification of existing measurements instruments for each domain or the development of new ones to measure the burden of neck pain.

## Introduction

Neck pain and low back pain are major sources of morbidity in many countries. In the Netherlands, neck pain and low back pain are the most common morbidities among the population aged 15–65 years old, outnumbered only by the incidence of infections of the respiratory system [1].

In order to assess the full impact on patients, health care and society it is important to have, besides numbers on prevalence and incidence, a full understanding of the burden of neck and back pain that takes into consideration the personal experience of people with the condition and their care providers. Recently a conceptual model of the burden of low back pain was developed [2]. A conceptual model is a type of diagram that shows the interrelationships between aspects or domains that are believed to constitute a construct. We do not know whether the conceptual models for burden of neck and burden of back pain are similar. In order to evaluate the effects of interventions on the burden of neck pain, valid and comprehensive outcome measures are needed, based on conceptual models that cover the full breadth of the construct.

A systematic review of measurement instruments for neck pain showed that of the eight neck-specific instruments, the Neck Disability Index (NDI) [3] was examined most for its measurement properties and it had the best methodological quality [4]. The NDI is a commonly used instrument to assess disability due to neck pain. It is a self-report questionnaire containing ten items on pain intensity, self-care, carrying, reading, headache, concentration, work, driving, sleep, and leisure [3]. A sum score, ranging from 0 to 50, is obtained by adding individual item scores, and higher scores reflect more limitations. In the Netherlands, the NDI is recommended by the Royal Dutch Society of Physical Therapy (KNGF) as the standard outcome measure for neck pain in the guidelines for treatment of whiplash [5]. A large number of studies have examined the dimensionality of the NDI using modern statistical approaches [6–10], and they have thrown some doubt on the unidimensionality. A content analysis of the NDI items also concludes that the items measure a broader concept than disability [11].

Concept mapping is a structured method for organizing the thoughts of a group [12–14]. It yields a concept map, i.e. a visual representation of all of the group’s thoughts relative to the specific topic and how these thoughts are connected [12]. It is particularly helpful in determining the individual elements of complex and unclear concepts. As it helps to ensure good content validity, it is very useful in defining constructs for the development of measurement instruments [2]. Content validity, as defined by Consensus-based Standards for the selection of health Measurement Instruments (COSMIN) [15], is ‘the degree to which the content of an instrument is an adequate reflection of the construct to be measured’. It is evaluated by judging the relevance of items with respect to the construct, the study population, and the purpose of the instrument (discriminative, evaluative or predictive) [15]. Relevance regarding both the construct and the study population is ensured by involving experts (i.e. persons with neck pain and their care providers) in the concept mapping groups.

The primary aim of this study was to empirically define the construct of burden of neck pain from the perspectives of both people with neck pain and healthcare providers with experience of managing them, using concept mapping. An additional aim of the study was to compare the conceptual model obtained for burden of neck pain with the content of the frequently used NDI.

## Methods

### Concept mapping

**Fig. 1:**
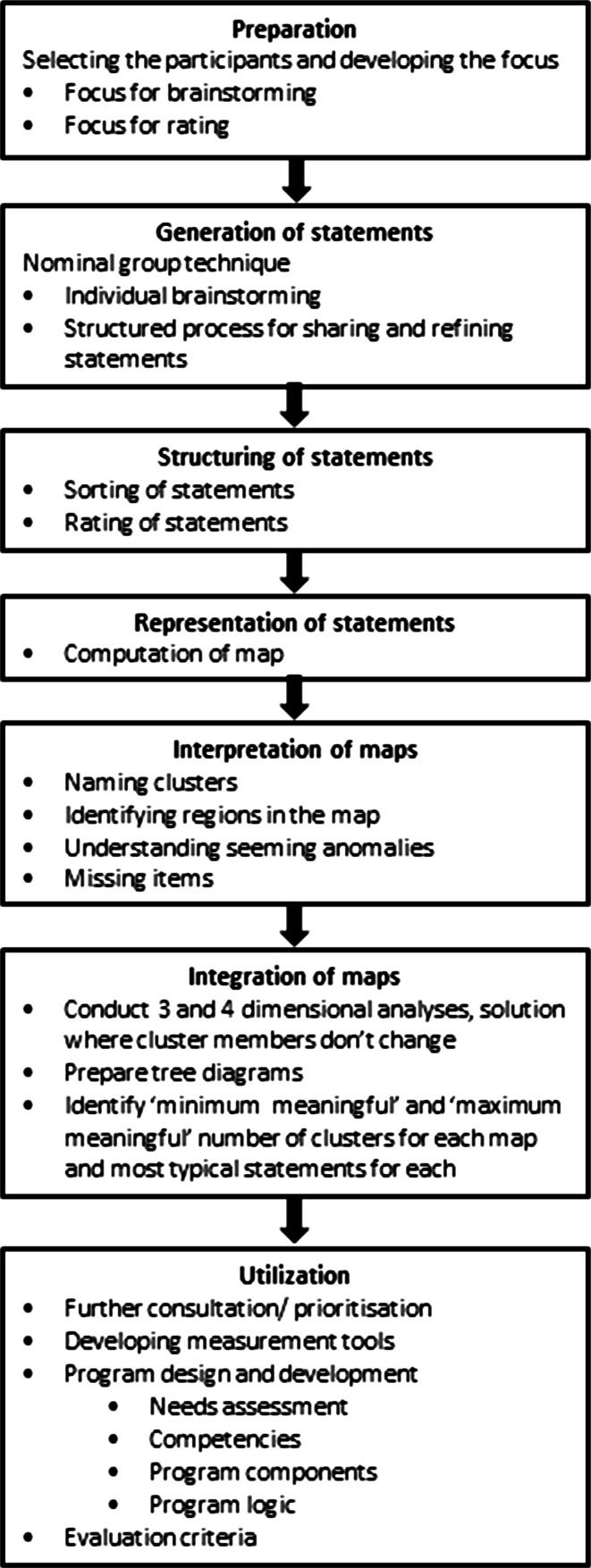
Steps of the concept mapping process, modified from Trochim [12]

The method of concept mapping consists of seven steps, as represented in Fig. 1 [12–14].

During the preparation phase (step 1), the seeding statement is formulated. In this study, the seeding statement for care providers was: ‘Thinking as broadly as you can, generate statements about how neck pain affects the life of people with neck pain and the people around them’, and the seeding statements for persons with neck pain was: ‘Thinking as broadly as you can, generate statements about how neck pain affects your life and the people around you’.

This seeding statement is the start for the generation of statements (step 2). Participants start by individually generating as many statements as they can think of in response to the seeding statement and they then share their statements within a nominal group process [16].

Once all ideas are presented, the structuring of statements (step 3) starts. Every participant is asked to individually sort the statements into conceptual similar groups in a way that makes sense to them and to name each group. After sorting the statements, participants rated the statements based on the question: ‘How *important* is this aspect of the burden of neck pain for persons with neck pain?’ A graphical representation of statements (step 4) is generated by computing a concept map. This is the quantitative part of the study. The concept map is computed based on the sorted data using non-metric multidimensional scaling (MDS) [17, 18] and on cluster analysis using Ward’s algorithm [19].

This concept map is interpreted (step 5) together with the participants who are then asked to name each cluster. After this group labelling process, the clusters are called domains. Furthermore, seeming anomalies are identified, which may lead to reallocation of items to other domains.

There are two ways to display data from the concept mapping: (1) in a concept map that emphasises the distances and relationships between ideas and (2) in a tree diagram, which allows exploration of the hierarchical nature of the data from broad concepts to more refined concepts and sub-concepts down to the individual items. SPSS was used to determine coordinates for individual two- and three-dimensional maps to use for MDS. Cluster analysis using Ward’s algorithm was then applied to the coordinates to produce a tree diagram showing all cluster solutions from three to 20 clusters. This allows the examination of the division of items each time a cluster is split into two smaller clusters. It is important to note that there is no correct number of clusters. As the aim of this study was a detailed and complete understanding of the burden of neck pain, it was important to continue the splitting process until we reached the maximum number of clusters that still makes sense to the researchers. Finally, the concept maps resulting from the groups with the persons with neck pain and the care providers were integrated (step 6) in a mind map, paying attention to the domain names given by the individual participants. If they were not too different, the concept maps for persons with neck pain and for care providers were integrated into one single mind map.

Step 7 concerns the utilization of the concept map and is not part of this project.

### Participants

We conducted five concept mapping groups: two with care providers and three with persons with neck pain. The first care providers group was recruited through the professional network of the EMGO Institute for Health and Care Research of the VU University Medical Center and consisted of six participants. All participants were experienced in both scientific research and the clinical management of neck pain. The meeting was held in English, because the Australian researchers (RHO and RWB) led this concept mapping group.

The second care providers group was recruited through e-mails and an announcement on the website of the Royal Dutch Society of Physiotherapists (KNGF), region Amstel, Meerlanden and Amsterdam (RGF AMA). Furthermore, physical therapy practices in Amsterdam were informed about the goal and the process of the concept mapping groups. The statement generation phase was performed by e-mail. Fifteen participants responded and 14 participants took part in this group. The language used in this group was Dutch (led by CvR-vdZ and HdV).

Concept mapping groups with persons with neck pain were performed in three different cities in the Netherlands. Inclusion criteria were a minimum age of 18, neck complaints as the main complaint and good comprehension of the Dutch language in order to be actively involved in the concept mapping process. The first workshop included persons with neck pain from one practice for physical and manual therapy in Zoetermeer (*n* = 5). The second included persons with neck pain from a variety of sources in Amsterdam (e.g. recruited by general practitioners and physical therapists) (*n* = 5). And the third included persons with neck pain from different physical therapy practices in the environment of Heerlen (*n* = 10). All group sessions for participants with neck pain were in Dutch and led by CvR-vdZ in the presence of HdV, AB, or RS. These four researchers, who attended concept mapping groups with both persons with neck pain and care providers, constructed the final mind map based on the five different concept maps.

The study protocol was evaluated and approved by the Ethics Committee of the VU University Medical Center in Amsterdam.

Finally, the correspondence between the items of the NDI [3] and the domains in the mind map was assessed by the researchers.

## Results

The number of statements generated in the separate concept mapping groups ranged from 46 to 68, and the total number of statements generated in five groups was 264. Of these statements, 118 were generated by care providers and 146 were generated by persons with neck pain.

**Fig. 2:**
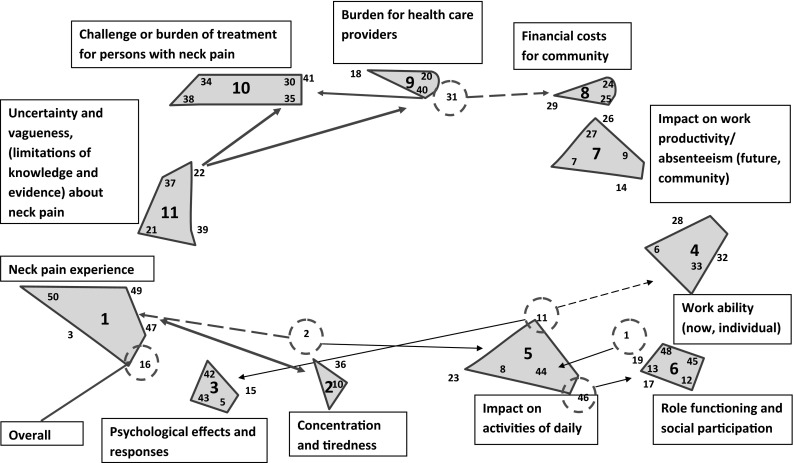
Concept map of first group with care providers. *Legend* The large numbers within the contoured forms (clusters) are assigned by the software as cluster number. The small numbers in and next to these clusters refer to the number of each statement belonging to that cluster. The statements *circled* by *dashed red lines* which are connected with a *solid arrow* to other clusters were considered to fit better in that other cluster (i.e. reallocated items at step 5). *Dashed arrows* represent a relation to the clusters they are associated with. The *text* in the *boxes* represent the names given to that cluster/domain. (Color figure online)

Figure 2 shows the concept map for the first group of care providers as an example. The interpretation is explained in the legend. There were 11 clusters, with the names given to domains presented in the boxes. The position of the various domains is determined by the closeness of the ideas. For example, in the right lower corner the domain ‘role functioning and social participation’ (cluster 6) is quite close to work ability (cluster 4), which is quite close to impact on work productivity and absenteeism (cluster 7). Furthermore, the domain ‘uncertainty and vagueness about neck pain’ (cluster 11) was linked with the clusters ‘challenge or burden of treatment for persons with neck pain’ and ‘burden for health care professionals’ (clusters 9 and 10).

The two concept maps for care providers were very similar, whereas the maps resulting from the three groups conducted with persons with neck pain were slightly different. One of these groups emphasized disagreement with care providers; the other group only mentioned uncertainty of diagnosis in general. The comparison of concept maps from the care providers and persons with neck pain revealed only minor differences. For example, the domain ‘difficult to treat/difficult to diagnose’ appeared in the care providers groups, whereas the persons with neck pain expressed this as uncertainty about diagnosis and treatments. The importance ratings of clusters, however, showed more differences between care providers and persons with neck pain: care providers rated all domains of neck pain as approximately equally important, while persons with neck pain rated ‘neck pain’, ‘accompanying and related complaints’, ‘activities of daily living’ (ADL) and ‘social participation’ as more important than the other domains.

**Fig. 3:**
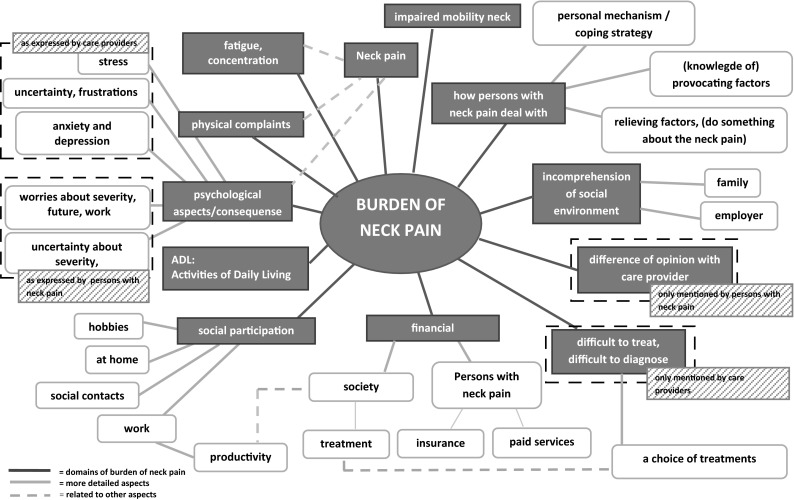
Integrated mind map of burden of neck pain

We decided to integrate the five concept maps into one mind map because the domains from the concept mapping groups conducted with care providers were very similar to those of persons with neck pain. The mind map as shown in Fig. 3 comprises 12 domains: impaired mobility neck, neck pain, fatigue/concentration, physical complaints, psychological aspects/consequences, ADL, social participation, financial consequences, difficult to treat/difficult to diagnose, difference of opinion with care providers, incomprehension by social environment, and how persons with neck pain deal with complaints. We summarized the domains fatigue/concentration, physical complaints, and psychological aspects/consequences as accompanying and related complaints. Physical complaints comprised complaints such as headache, dizziness, radiating pain in shoulders and arm, and loss of strength. ADL included activities such as cycling/driving, computer tasks, household duties, sleeping, reading, and self-care. Statements that comprised relieving factors included ‘warmth relieves the neck pain’, ‘paying attention to posture leads to less pain in the neck’, ‘adaptations are needed, e.g. foot mouse, screen glasses, cushion’, ‘variation in posture and activities’, ‘the use of pain medication’, and ‘occasional treatment by therapist’. Personal mechanism/coping strategy comprised statements such as ‘deal with the pain’, ‘distract yourself from the pain by undertaking lots of other activities’, ‘don’t express the fact that neck hurts’, ‘wanting to rise above yourself’, and ‘show others that you are able to do things’. (Knowledge of) provocating factors included statements such as ‘sitting/standing for a long period of time increases neck pain’, ‘repetitive movements lead to neck pain’, and ‘avoid lifting heavy objects’.

The mind map reflects that neck pain influences fatigue/concentration, physical complaints, and psychological aspects/consequences, but then these three domains also influence neck pain. The concept mapping groups revealed that it is sometimes hard to distinguish cause from consequence and that these aspects may tend to be a vicious circle. Furthermore, financial consequences were split into consequences for the individual and for society, where financial consequences are linked to productivity and treatment-related costs.

Originally, the NDI is intended to measure ‘self-rated disability’. When assessing all ten separate items of the NDI, it is possible to link all ten items to our mind map. The NDI items personal care, reading, driving, and sleep were mentioned in the domain ‘ADL’; the NDI item lifting was captured in the domain ‘how persons with neck pain deal with complaints’, specifically in the domain ‘(knowledge of) provocating factors’; the NDI items headache and concentration were included in the accompanying and related complaints; and the NDI items work and recreation were captured by the domain ‘social participation’. The NDI item on pain intensity corresponds with neck pain, although pain intensity was not explicitly mentioned in the concept mapping groups. Conversely, the NDI lacks ideas such as ‘psychological aspects/consequences’, ‘financial consequences’, ‘difficult to treat/difficult to diagnose’, ‘difference of opinion with health professional’, incomprehension by social environment’, and ‘how persons with neck pain deal with pain’.

## Discussion

This is the first study to systematically conceptualize the burden of neck pain that involves both persons with neck pain and healthcare providers with experiences of managing them. The integration of concept maps resulting from the five concept mapping groups led to the mind map in Fig. 3. By combining the five concept maps, all domains of the burden of neck pain, from the perspectives of persons with neck pain as well as healthcare providers, are preserved. The importance ratings of clusters reveal which domains of burden of neck pain are more important than others for persons with neck pain. This is useful information to help decide the focus of interventions and for determining which domains should be included in the measurement of burden of neck pain. All ten items of the NDI could be linked to domains in the mind map, but some domains were not represented by NDI items.

The combined results from the five concept mapping groups revealed 12 domains for ‘burden of neck pain’. The conceptual model for the burden of low back pain [2] showed large similarities to our conceptual model for the burden of neck pain. The burden of low back pain contains six domains, which are named: ‘physical’, ‘psychological’, ‘social’, ‘employment’, ‘treatment’, and ‘positive effects’. Some of these domains are divided into subdomains. The ‘physical’ domain of burden of low back pain corresponds to some extent with our accompanying and related complaints and the domains ‘ADL’ and ‘social participation’ of burden of neck pain. The model of burden of low back pain has a separate domain ‘psychological’, which is interwoven with the domains ‘accompanying and related complaints’ and ‘how persons deal with pain’ in the neck pain model. Furthermore, the domain ‘social’ of the burden of back pain model is captured by both ‘social participation’ and ‘incomprehension by social environment’ in our model of neck pain. In summary, the burden of low back pain is to great extent comparable to the burden of neck pain, although there are some minor differences. This raises the question of the feasibility of proposing one single model for chronic pain instead of different models for chronic pain of various conditions. Future studies using concept mapping in populations with other chronic pain conditions might reveal which domains are common and which are distinct.

As the developers of the NDI did not start from a clear definition of the concept to be measured, it is unclear what the NDI measures exactly [11]. We showed that the NDI items refer to different domains within the conceptual model for burden of neck pain, but not all domains are represented in the NDI. Some studies have shown the multidimensionality of the NDI [7], and some even propose to delete items in order to reach unidimensionality [6, 8–10]. A recent study on exploratory factor analysis of the NDI [11] showed that two factors (physical and non-physical) could be identified that explain 53.8 % of the variance. The non-physical factor was difficult to interpret as it contained only the NDI items reading, headaches, and concentration. Considering the fact that the NDI is not unidimensional and all NDI items can be linked to some of the domains in our model of burden of neck pain, it can be concluded that the NDI measures a broader concept than disability.

The next step in adequately measuring the burden of neck pain is identifying comprehensive questionnaires or subscales of questionnaires to measure the different domains. It is not necessary to have one single measurement instrument to measure the whole concept of burden of neck pain; separate instruments can be used for separate domains. The scales are preferably unidimensional for each domain. The burden of neck pain mind map gives a clear overview of the relevant domains of the burden of neck pain and will guide the selection of existing scales or the development of new scales for all of these constructs. The statements generated in the concept mapping groups also provide plain language statements, grouped by domain, that make a useful starting point to draft items. This is another important advantage of concept mapping.

A few limitations apply to this study. First of all, we did not gather extensive demographic and clinical data on the participants, including specific characteristics of their neck pain. Therefore, we do not know whether our participants were representative of all persons with neck pain. The concept mapping groups included mainly persons with chronic neck pain. This implies that the conceptual model will better reflect the burden of chronic neck pain than the burden of acute neck pain. However, persons with chronic neck pain experience a wide variety of different complaints for a longer period of time, with consequences in many domains of their daily lives and we can therefore assume that their burden is more diverse than the burden for persons with acute neck pain. In other words, a concept map for persons with chronic neck pain probably captures most aspects that would be revealed in a concept map for persons with acute neck pain, while the reverse is unlikely. This hypothesis can be tested by repeating this research project among persons with acute neck pain.

Second, the majority of the persons with neck pain were recruited by their physical therapist and only few were recruited by general practitioners or other care providers. This is in line with healthcare practice in the Netherlands where most persons with neck pain are treated by physical therapists.

Only researchers were involved in the process of integrating the five concept maps into the final mind map, no persons with neck pain. We felt that being present at two or more focus groups (at least one with persons with neck pain and one with care providers) was necessary to experience the full scope of the discussions—including the atmosphere and context in which things were said—and to be able to integrate the five concept maps into one mind map. However, feedback from persons with neck pain on the final mind map would have been interesting.

In conclusion, by combining both qualitative and quantitative research methods, concept mapping revealed a wide range of domains representing the burden of neck pain. The results of this study are a good starting point to understand the gaps in neck pain outcome measurement. From here, we can move forward by either identifying existing measurement instruments for each domain or developing new ones.
